# Inner retinal thinning as a biomarker for cognitive impairment in *de novo* Parkinson’s disease

**DOI:** 10.1038/s41598-019-48388-7

**Published:** 2019-08-14

**Authors:** Mi Sun Sung, Seong-Min Choi, Jonghwa Kim, Jun Young Ha, Byeong-Chae Kim, Hwan Heo, Sang Woo Park

**Affiliations:** 1 Department of Ophthalmology, Chonnam National University Medical School and Hospital, Gwangju, South Korea; 2 Department of Neurology, Chonnam National University Medical School and Hospital, Gwangju, South Korea

**Keywords:** Diagnostic markers, Parkinson's disease

## Abstract

We investigated the association between retinal changes measured using optical coherence tomography (OCT) and diverse clinical grading scales in patients with Parkinson’s disease (PD). Seventy-four eyes of 74 patients with *de novo* PD and 53 eyes of age-matched control subjects were included. The thickness of the peripapillary retinal nerve fiber layer (pRNFL) and macular ganglion cell-inner plexiform layer (mGCIPL) were measured. We analyzed the correlations between the clinical PD grading scales and OCT parameters, and between the OCT parameters and volumetric data in the cerebral cortical and subcortical structures. The area under the receiver operating characteristic curve (AUC) was calculated for diagnosing cognitive impairment in patients with PD. Statistically significant reductions in the thickness of average, temporal, and inferior pRNFL and overall mGCIPL were observed in patients with PD. The Montreal Cognitive Assessment score was significantly associated with mGCIPL thinning. The AUC of the mGCIPL parameters for diagnosing cognitive impairment in patients with PD ranged from 0.651 to 0.760. Moreover, thinning of the mGCIPL was significantly associated with the volumetric parameters of associated brain structures. Our findings highlight the clinical implications of OCT measurements as a potential biomarker for early detection of cognitive impairment in patients with PD.

## Introduction

Parkinson’s disease (PD) is the second most common neurodegenerative disorder in which the main pathologic changes occur in the dopaminergic neurons of substantia nigra^1^. Patients with PD experience a broad spectrum of motor and non-motor symptoms, such as bradykinesia, rigidity, resting tremor, cognitive impairment, hallucinations, depression, sleep disorders, and autonomic dysfunctions. Recently, the role of non-motor symptoms has gained relevance, because they influence the course of PD and contribute substantially to the disease burden^2^. Among these, cognitive impairment is significantly associated with a poor quality of life. Mild cognitive impairment (MCI) is highly prevalent and is a risk factor for PD with dementia^3^.

Recently, studies regarding biomarkers other than brain imaging have gained particular interest to provide reliable indicators of clinical manifestations in patients with PD. The retina is considered an extension of the central nervous system (CNS) and has gained attention in the research of neurodegenerative disorders^4,5^. In ophthalmic research, spectral domain-optical coherence tomography (SD-OCT) provides a non-invasive, objective, and reproducible measurement of retinal structures of the eye^6^. Retinal changes measured by OCT have been considered as useful biomarkers in several neurodegenerative diseases, including PD. Previous studies have reported a significant reduction in the thickness of the peripapillary retinal nerve fiber layer (pRNFL) in patients with PD compared to normal controls^7,8^. Similarly, a reduction in the macular thickness has been reported in patients with PD^9–12^. However, there are limited studies on the clinical correlation of OCT parameters in patients with PD; therefore, the significance of retinal measurement in clinical settings remains controversial.

Evaluation of patients with PD is performed using diverse clinical grading scales. The modified Hoehn and Yahr (mHY) scale, which reflects the severity of the motor impairment and balance/gait compromise, and the Unified Parkinson’s Disease Rating Scale (UPDRS) parts II and III, which assess the activities of daily living and motor impairment, are the commonly used scales for assessing the motor symptoms in patients with PD^13,14^. In addition, various non-motor scales are used for the assessment of each non-motor symptom of PD^15^. The current study investigated the association between the retinal changes measured by SD-OCT and various clinical grading scales evaluating motor and non-motor manifestations in patients with PD. In addition, the volume of the cerebral cortex and subcortical structures were measured using automated segmentation methods, and the correlation between these volumetric data and OCT parameters were investigated to determine the clinical significance of retinal measurements in patients with PD^16,17^.

## Results

A total of 82 patients with PD were enrolled in the present study. Three subjects were excluded due to glaucomatous optic nerve head (ONH) changes, and five due to unacceptable quality of the OCT images. Finally, 74 eyes of 74 patients with PD and 53 eyes of 53 controls were included in the analysis. A summary of the demographic variables and OCT measurements of the subjects is shown in Table 1. The age of the participants was 65.30 ± 8.38 years and 64.68 ± 6.64 years for the PD and control group, respectively. No significant between-group differences were observed in the best corrected visual acuity (BCVA), spherical equivalent (SE) refractive error, intraocular pressure (IOP), axial length, central corneal thickness, optic disc area, and the vertical cup-to-disc (CDR) ratio. The average mHY scale score in patients with PD was 1.76 ± 0.76 (Table 1). Overall, the OCT measurements pertaining to thickness of the pRNFL, macula, and macular ganglion cell-inner plexiform layer (mGCIPL) showed significant differences between the two groups. The differences were more pronounced in the thickness of the mGCIPL; however, all parameters were significantly smaller in the PD group than in the control group (*P* < 0.05) (Table 2).

**Table 1:** Demographic characteristics of the study participants.

Characteristics	Parkinson’s disease (n = 74)	Normal controls (n = 53)	*P*-value^*^
Age (years)	65.30 ± 8.38	64.68 ± 6.64	0.657
Sex (male: female)	25:49	18:35	0.983
BCVA (logMAR)	0.05 ± 0.08	0.03 ± 0.04	0.136
SE refractive error (D)	0.51 ± 1.38	0.12 ± 0.94	0.113
IOP (mmHg)	14.80 ± 3.07	15.11 ± 1.98	0.482
Axial length (mm)	23.66 ± 1.12	23.81 ± 1.27	0.231
Central corneal thickness (μm)	526.11 ± 42.04	531.26 ± 53.19	0.336
Disc area (mm^2^)	2.11 ± 0.42	2.15 ± 0.48	0.618
Vertical CDR	0.54 ± 0.14	0.57 ± 0.15	0.187
Neurologic examinations			
mHY scale	1.76 ± 0.76	—	—
UPDRS part I	1.76 ± 2.11	—	—
UPDRS part II	6.37 ± 5.26	—	—
UPDRS part III	18.78 ± 8.74	—	—
NMSS	42.97 ± 34.78	—	—
BDI	34.03 ± 13.30	—	—
PDQ-8	4.89 ± 4.94	—	—
K-MMSE	25.69 ± 3.50	—	—
MoCA	23.90 ± 4.88	—	—

BCVA = best-corrected visual acuity; SE = spherical equivalent; D = diopters; IOP = intraocular pressure; CDR = cup-to-disc ratio; mHY = modified Hoehn & Yahr scale; UPDRS = United Parkinson’s Disease Rating Scale; NMSS = Non-Motor Symptom Scale; BDI = Beck Depression Inventory; PDQ-8 = Parkinson’s disease quality of life 8 questions; K-MMSE = Korea version of the Mini-Mental State Examination; MoCA = Montreal Cognitive Assessment.

Data are expressed as mean ± standard deviation unless otherwise indicated.

^*^P-values derived from Student-*t* test or Chi-square test as appropriate.

**Table 2:** Comparison of OCT measurements between patients with Parkinson’s disease and normal controls.

	Parkinson’s disease (n = 74)	Normal controls (n = 53)	*P*-value^*^
pRNFL thickness (μm)			
Average	90.74 ± 9.88	95.71 ± 7.02	**0.001**
Superior	115.09 ± 16.32	119.69 ± 12.26	0.091
Inferior	116.11 ± 24.26	124.55 ± 13.63	**0.015**
Nasal	65.65 ± 10.65	66.92 ± 7.44	0.462
Temporal	64.69 ± 10.00	71.62 ± 8.74	**<0.001**
mGCIPL thickness (μm)			
Minimum	73.30 ± 9.01	79.30 ± 5.14	**<0.001**
Average	78.69 ± 6.68	82.75 ± 4.51	**<0.001**
Superonasal	80.74 ± 8.06	83.94 ± 5.00	**0.007**
Superior	80.66 ± 8.55	83.00 ± 5.58	**0.048**
Superotemporal	78.43 ± 6.68	81.72 ± 5.34	**0.004**
Inferotemporal	78.54 ± 7.34	82.49 ± 6.51	**0.002**
Inferior	76.16 ± 8.50	80.51 ± 5.99	**0.001**
Inferonasal	77.91 ± 8.12	82.28 ± 5.56	**<0.001**
CFT (μm)	240.00 ± 24.49	255.34 ± 45.90	**0.016**
Average MT (μm)	274.15 ± 13.48	279.96 ± 11.86	**0.013**
Overall MV (mm^3^)	9.83 ± 0.49	10.00 ± 0.42	**0.034**

pRNFL = peripapillary retinal nerve fiber layer; mGCIPL = macular ganglion cell-inner plexiform layer; CFT = central foveal thickness; MT = macular thickness; MV = macular cube volume.

Data are mean ± standard deviation unless otherwise indicated.

Factors with statistical significance are shown in boldface.

^*^P-values derived from Student-*t* test.

The relationship between the various clinical grading scales of PD and thickness parameters measured by SD-OCT is presented in Table 3. Generally, PD scales associated with the motor symptoms, mHY scale, UPDRS parts II and III, had no significant association with the OCT measurements. Conversely, the Non-Motor Symptom Scale (NMSS), used to assess the non-motor symptoms of PD, showed a significant correlation with the average pRNFL (*P* = 0.043), and the minimum, average, inferotemporal, inferior, and inferonasal thickness of mGCIPL (*P* = 0.004, *P* = 0.006, *P* = 0.002, *P* < 0.001, and *P* = 0.023, respectively). In particular, PD scales representing the cognitive function, such as the Korean version of the Mini-Mental State Examination (K-MMSE) and Montreal Cognitive Assessment (MoCA) scale were found to have a more significant correlation with all parameters of thickness of the mGCIPL (*P* < 0.05). Although the whole macular thickness parameters, such as central foveal thickness (CFT), average macular thickness (MT), and overall macular cube volume (MV) also showed a significant association with the clinical grading scales in patients with PD, the strength of the association was weaker than that of the mGCIPL thickness parameters.

**Table 3:** Correlation between the structural OCT measurements and various clinical grading scales in patients with Parkinson’s disease.

	mHY scale	UPDRS part I	UPDRS part II	UPDRS part III	NMSS	BDI	PDQ-8	K-MMSE	MoCA
pRNFL thickness									
Average	0.027 (0.818)	0.017 (0.920)	−0.109 (0.379)	−0.084 (0.478)	−**0.244 (0.043)**	−0.130 (0.286)	−0.101 (0.554)	0.169 (0.151)	0.193 (0.117)
Superior	−0.023 (0.843)	0.188 (0.259)	−0.227 (0.065)	−0.195 (0.095)	−0.061 (0.621)	−0.120 (0.327)	0.076 (0.655)	0.191 (0.103)	0.168 (0.174)
Inferior	−0.044 (0.712)	−0.151 (0.365)	−0.012 (0.992)	−0.024 (0.841)	−0.223 (0.066)	−0.103 (0.402)	−0.285 (0.087)	0.075 (0.528)	0.184 (0.136)
Nasal	0.179 (0.076)	−0.126 (0.450)	−0.158 (0.201)	0.141 (0.232)	−0.179 (0.141)	−0.114 (0.350)	0.065 (0.701)	0.026 (0.826)	−0.181 (0.144)
Temporal	0.060 (0.611)	0.261 (0.114)	0.152 (0.140)	0.069 (0.559)	−0.117 (0.337)	0.023 (0.848)	0.057 (0.736)	0.079 (0.506)	0.056 (0.654)
mGCIPL thickness									
Minimum	−0.227 (0.051)	−0.031 (0.516)	−0.088 (0.481)	−0.161 (0.169)	**−0.339 (0.004)**	−0.113 (0.357)	−0.141 (0.406)	**0.387 (0.001)**	**0.505 (<0.001)**
Average	−0.153 (0.194)	−0.031 (0.852)	−0.056 (0.653)	−0.086 (0.466)	**−0.329 (0.006)**	−0.103 (0.400)	−0.178 (0.292)	**0.346 (0.003)**	**0.471 (<0.001)**
Superonasal	−0.121 (0.304)	−0.066 (0.696)	−0.024 (0.845)	−0.055 (0.642)	−0.244 (0.054)	−0.094 (0.441)	−0.034 (0.839)	**0.355 (0.002)**	**0.440 (<0.001)**
Superior	−**0.257 (0.027)**	−0.117 (0.484)	0.027 (0.828)	−0.165 (0.160)	−0.179 (0.141)	−0.053 (0.667)	0.024 (0.887)	**0.319 (0.006)**	**0.479 (<0.001)**
Superotemporal	−0.096 (0.414)	−0.069 (0.682)	0.050 (0.690)	0.040 (0.735)	−0.156 (0.200)	0.008 (0.948)	−0.115 (0.498)	**0.247 (0.034)**	**0.359 (0.003)**
Inferotemporal	−0.095 (0.419)	−0.163 (0.329)	−0.072 (0.565)	0.047 (0.693)	**−0.366 (0.002)**	0.003 (0.982)	**−0.432 (0.008)**	**0.275 (0.018)**	**0.483 (<0.001)**
Inferior	−0.152 (0.196)	−0.077 (0.644)	−0.171 (0.166)	−0.074 (0.530)	**−0.423 (<0.001)**	−0.125 (0.308)	−0.262 (0.117)	**0.333 (0.004)**	**0.416 (<0.001)**
Inferonasal	−0.120 (0.310)	−0.009 (0.956)	−0.108 (0.382)	−0.092 (0.437)	**−0.273 (0.023)**	−0.081 (0.508)	−0.052 (0.758)	**0.349 (0.002)**	**0.412 (0.001)**
CFT	−0.016 (0.894)	−0.168 (0.314)	−0.062 (0.620)	−0.030 (0.797)	0.104 (0.394)	−0.065 (0.597)	0.091 (0.591)	0.126 (0.283)	−0.095 (0.446)
Average MT	−0.080 (0.496)	−0.205 (0.216)	−0.136 (0.271)	−0.076 (0.520)	**−0.292 (0.015)**	−0.008 (0.226)	−0.158 (0.349)	**0.299 (0.010)**	**0.331 (0.006)**
Overall MV	−0.077 (0.516)	−0.177 (0.287)	−0.149 (0.227)	−0.071 (0.547)	**−0.293 (0.015)**	−0.006 (0.216)	−0.132 (0.434)	**0.282 (0.013)**	**0.345 (0.004)**

pRNFL = peripapillary retinal nerve fiber layer; mGCIPL = macular ganglion cell-inner plexiform layer; CFT = central foveal thickness; MT = macular thickness; MV = macular cube volume; mHY = modified Hoehn & Yahr scale; UPDRS = United Parkinson’s Disease Rating Scale; NMSS = Non-Motor Symptom Scale; BDI = Beck Depression Inventory; PDQ-8 = Parkinson’s disease quality of life 8 questions; K-MMSE = Korea version of the Mini-Mental State Examination; MoCA = Montreal Cognitive Assessment.

Values are expressed as Pearson correlation coefficients (*P* values in parentheses).

Factors with statistical significance are shown in boldface.

**Figure 1:**
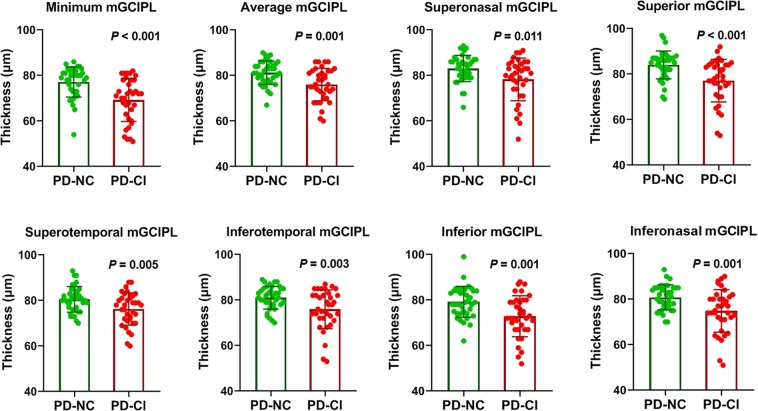
Comparison of the mGCIPL thickness between the patients with PD who had a normal cognition (PD-NC) and those with a cognitive impairment (PD-CI). PD = Parkinson’s disease; mGCIPL = macular ganglion cell-inner plexiform layer.

**Figure 2:**
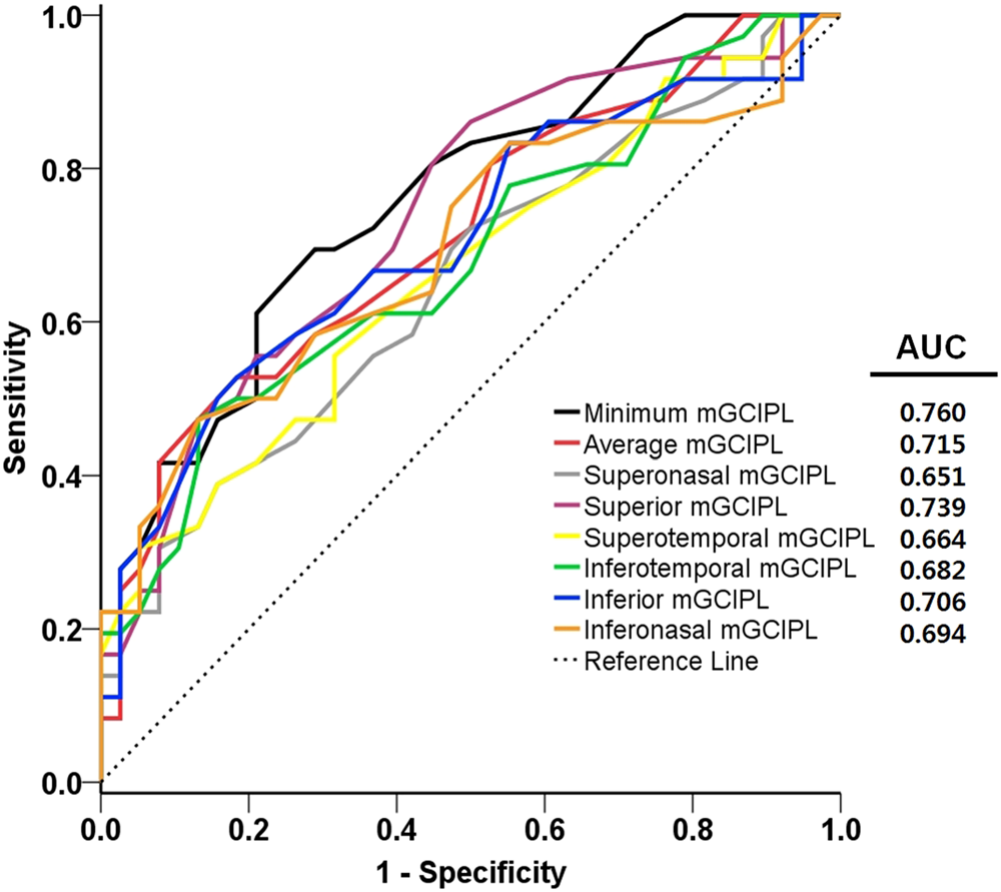
ROC curves and AUC values of the mGCIPL thickness parameters for detection of cognitive impairment in patients with PD. ROC = receiver operating characteristic; AUC = area under the ROC curve; mGCIPL = macular ganglion cell-inner plexiform layer; PD = Parkinson’s disease.

According to previous reports^18,19^, MoCA scores equal to or less than 25 represent adequate psychometric properties as a screening instrument for detection of mild cognitive impairment in patients with PD. When patients with PD were further divided into two groups, those with normal cognition (PD-NC, n = 38) and those with cognitive impairment (PD-CI, n = 36), the patients in the PD-CI group demonstrated significantly reduced thickness of the mGCIPL compared to those in the PD-NC group (minimum, 69.22 ± 9.45 vs. 77.16 ± 6.65 μm; average, 76.00 ± 7.11 vs. 81.24 ± 5.16 μm; superonasal, 78.28 ± 9.38 vs. 83.08 ± 5.78 μm; superior, 77.11 ± 9.36 vs. 84.03 ± 6.12 μm; superotemporal, 76.25 ± 7.10 vs. 80.50 ± 5.60 μm; inferotemporal, 75.92 ± 8.49 vs. 81.03 ± 5.01 μm; inferior, 72.89 ± 8.98 vs. 79.26 ± 6.78 μm; inferonasal, 74.83 ± 9.31 vs. 80.82 ± 5.49 μm, respectively) (Fig. 1). To determine the value of OCT measurements in the diagnosis of cognitive impairment in patients with PD, receiver operating characteristics (ROC) curves were drawn with a cut-off value of MoCA ≤25. The area under the ROC curve (AUC) was the highest in cases of minimum thickness of the mGCIPL (AUC = 0.760). All mGCIPL measurements showed a favorable, fine diagnostic power with an AUC of more than 0.650 (Fig. 2).

For the correlation analysis between the OCT parameters and volumetric data of the cerebral cortex and subcortical structures, 37 patients with PD who underwent brain magnetic resonance imaging (MRI) at Chonnam National University Hospital were additionally evaluated. The total intracranial volume and the volumes of cerebellar gray matter (GM), cerebellar white matter (WM), and caudate nucleus did not show any significant association with the mGCIPL parameters. However, the volumes of cortical WM and GM, thalamus, putamen, pallidum, and hippocampus showed mild-to-moderate correlations with several thickness parameters of the mGCIPL. Among the brain structures, the volumes of cortical WM and thalamus showed stronger relationships with the thickness of the mGCIPL (Table 4).

**Table 4:** Correlation between the mGCIPL thickness and brain volumes measured using an automated segmentation method (FreeSurfer) in patients with PD.

	Minimum	Average	Superonasal	Superior	Superotemporal	Inferotemporal	Inferior	Inferonasal
Intracranial volume	−0.053 (0.755)	0.022 (0.899)	−0.112 (0.509)	0.001 (0.998)	0.006 (0.970)	0.089 (0.599)	−0.037 (0.826)	−0.184 (0.275)
Cortical GM	**0.423 (0.009)**	0.219 (0.193)	**0.390 (0.017)**	0.162 (0.338)	0.303 (0.068)	0.314 (0.059)	**0.433 (0.007)**	**0.520 (0.001)**
Cortical WM	**0.496 (0.002)**	**0.378 (0.021)**	**0.453 (0.005)**	0.232 (0.167)	0.220 (0.191)	**0.408 (0.012)**	**0.556 (<0.001)**	**0.573 (<0.001)**
Cerebellar GM	0.204 (0.226)	0.008 (0.961)	0.138 (0.417)	−0.095 (0.576)	0.100 (0.554)	0.267 (0.110)	0.339 (0.092)	0.314 (0.158)
Cerebellar WM	0.297 (0.074)	0.228 (0.175)	0.250 (0.136)	0.074 (0.664)	0.148 (0.382)	0.295 (0.076)	0.301 (0.108)	0.297 (0.165)
Thalamus	**0.513 (0.001)**	**0.328 (0.047)**	**0.456 (0.005)**	0.239 (0.155)	**0.469 (0.003)**	**0.446 (0.006)**	**0.496 (0.002)**	**0.571 (<0.001)**
Putamen	**0.396 (0.015)**	0.310 (0.062)	**0.396 (0.015)**	0.268 (0.109)	0.293 (0.078)	0.305 (0.066)	**0.343 (0.038)**	**0.388 (0.018)**
Pallidum	**0.425 (0.009)**	**0.370 (0.024)**	**0.441 (0.006)**	0.239 (0.153)	0.280 (0.093)	0.254 (0.129)	**0.394 (0.016)**	**0.548 (<0.001)**
Caudate nucleus	−0.253 (0.132)	−0.302 (0.063)	−0.244 (0.146)	−0.289 (0.083)	−0.124 (0.466)	−0.091 (0.593)	−0.237 (0.158)	−0.241 (0.151)
Hippocampus	**0.354 (0.032)**	0.219 (0.193)	0.324 (0.050)	0.151 (0.372)	0.205 (0.223)	0.238 (0.157)	**0.344 (0.037)**	**0.496 (0.002)**

mGCIPL = macular ganglion cell-inner plexiform layer; GM = gray matter; WM = white matter.

Values are expressed as Pearson correlation coefficients (*P* values in parentheses).

Factors with statistical significance are shown in boldface.

## Discussion

PD is the second most common neurodegenerative disorder, with a gradual increase being observed in the prevalence of the condition^20^. The diagnosis and monitoring of PD rely mainly on the clinical manifestations, motor and non-motor symptoms. However, the clinical manifestations of PD are often difficult to diagnose in the early stages, since symptoms occur when as many as 50% of the dopaminergic nigral neurons have already been lost. Moreover, a direct assessment of the clinical symptoms is difficult. Therefore, much progress has been made in investigating the objective biomarkers of PD and translating these into useful tools for management of patients with PD.

Clinically, biomarkers can be used for indicating susceptibility, early detection of a disease, monitoring of a disease’s progression, and evaluation for therapeutic interventions. Several biomarkers have been suggested, including biofluids, peripheral tissues, genetic, and imaging, to monitor the onset and progression of the clinical manifestations of PD. Lower levels of cerebrospinal fluid (CSF) α-synuclein and amyloid-β, and increased level of blood triglycerides have been reported to be associated with the clinical manifestations of PD^21,22^. Although controversial, the presence of aggregated α-synuclein in the skin and gastrointestinal tract has been reported to be a prodromal finding for the development of PD^23^. Through an understanding of the genetic factors in the development of PD, different gene expression patterns have been reported^24^. In addition, several structural, functional, and molecular imaging modalities have also been widely applied in the search for biomarkers for PD^25^. Collectively, the preliminary promising aspects of these biomarkers await further validation prior to their use as reproducible and reliable biomarkers of PD.

The prominent pathologic change of PD is the selective degeneration of dopaminergic neurons in the substantia nigra^1^. Dopaminergic neurons are also present in areas such as the retinal ganglion cells (RGCs) and amacarine cells in the retina^26^. A previous histological study reported a decreased concentration of dopamine in the eye in patients with PD^27^. PD could also present with visual symptoms, such as reduced contrast sensitivity, motion perception abnormalities, and color deficiencies^28^. Based on these findings, the association of retinal changes with PD has been widely investigated in the field of study of biomarkers for PD. The recent development of OCT technology has made it possible to efficiently image and quantify the retinal microstructure with increased precision. Recent studies using SD-OCT have described a significant reduction in the inner retinal thickness in patients with PD^7–12,29,30^. The inner retinal thickness reflects the RGCs, nerve fiber layer (axons of RGCs), and inner plexiform layer. In accordance with previous findings, a decreased thickness of the pRNFL, macula, and mGCIPL were observed in patients with PD compared with normal controls in the current study, and the thinning was more prominent in the mGCIPL. Although the percentage of dopaminergic neurons is quite small in the inner retina and they are mostly amacrine cells localized in the inner nuclear layer and are not a part of the ganglion cell layer, we speculated that the progressive retinal dopaminergic deficiency causes loss of the retinal amacrine cells that provide input to the RGCs, thereby causing mGCIPL thinning in patients with PD.

Regarding the pattern of inner retinal thinning, studies have reported that the loss of RGCs in PD preferentially involves the temporal quadrant of the pRNFL^29–31^. A study by La Morgia *et al*.^29^ reported a thinner temporal pRNFL compared to controls. Similarly, in the current study, we also observed a non-uniformity in the thinning of the pRNFL, which was more pronounced in the temporal and inferior quadrants compared to the superior and nasal quadrants. This may be explained by the fact that PD mainly affects the papillomacular bundle (parvocellular RGC axons)^29,31^. Owing to the inferior position of the macula relative to the optic disc, the papillomacular bundles lead to the temporal and inferior quadrants of the optic disc; thus, a damage to this bundle causes thinning of the temporal and inferior pRNFL^32^. The more pronounced thinning of the mGCIPL compared to the pRNFL might also be attributable to the same reason. The parvocellular cells are more vulnerable to mitochondrial dysfunction, and the pattern of loss of RGCs observed in patients with PD resembles that of mitochondrial optic neuropathies, such as the Leber’s hereditary optic neuropathies and dominant optic atrophy. It has been postulated that α-synuclein deposition and mitochondrial dysfunction in the retina in PD may ultimately lead to a prevalent damage of the parvocellular cells^31^.

Previous studies have demonstrated a significant association between the inner retinal thickness and the severity of PD^8,10–12,33^. Garcia-Martin *et al*.^12,33^ reported that the ganglion cell layer and the thickness of the pRNFL were inversely correlated with the duration and severity of the motor symptoms of PD (HY scale). However, the current study did not reveal significant associations between the inner retinal thickness and the severity of the motor symptoms in PD, as measured using the mHY scale and UPDRS part III. This discrepancy could be attributed to the differences in the study populations; the population with PD in the current study comprised of drug-naïve patients at a relatively early stage of the disease, with mild motor symptoms (mean mHY score of 1.76 ± 0.76 and mean UPDRS part III of 18.78 ± 8.74). The current study demonstrated that patients with PD who had a greater damage of the mGCIPL presented with more severe non-motor symptoms and cognitive impairment. The average and minimum thickness, as well as the thickness of all six sectors of the mGCIPL significantly correlated with the K-MMSE and MoCA scores. The relatively high AUC values of the mGCIPL parameters indicate that OCT might be a useful tool for differentiating the patients with cognitive impairment among populations with PD.

In this study, the whole macular thickness parameters, such as CFT, average MT, and overall MV, also showed a significant association with the cognitive impairment in patients with PD. Considering that the strength of the associations between the macular parameters and clinical PD grading scales was weaker than that of the mGCIPL thickness parameters, we suggest that the thinning of the mGCIPL might have affected the result of the correlation analysis, because the whole macular thickness measurements include the inner retinal layer. In a recent report, Uchida *et al*.^34^ demonstrated that there were no identifiable changes in the outer retinal metrics in patients with a neurodegenerative disease, such as PD, which might support our assumption. Future investigations regarding the structural changes of other retinal layers in PD would provide more valuable information about the role of OCT in patients with PD.

Cognitive impairment at baseline is one of the most consistent risk factors of dementia in patients with PD^35^. Therefore, the early identification of cognitive impairment in patients with PD is of particular importance. Regarding biomarkers of cognitive impairment, Parnetti *et al*.^36^ reported that the reduced levels of CSF amyloid-β 42 represented a predictive factor for cognitive deterioration, and lower CSF amyloid-β 42 level was associated with a higher rate of decline in MMSE and MoCA scores in patients with PD. Lindqvist *et al*.^37^ also reported a significant correlation of the MMSE score with the CSF interleukin-6 levels. Additionally, one study reported that subcortical atrophy was associated with the cognitive impairment in patients with mild PD^38^. However, the use of these markers is hampered by the lack of sensitivity during the early stages of the disease, high cost, and invasiveness. In this regard, the findings of the current study are clinically important owing to the demonstration of the potential of OCT measurement, which is non-invasive and cost-effective, as a diagnostic marker for cognitive impairment in patients with PD.

The pathogenesis of cognitive impairment in PD is complex and involves multiple factors. The process might be driven by a disruption of the dopaminergic, cholinergic, and serotonergic neurotransmitter systems secondary to the aggregation of abnormal α‐synuclein; however, amyloid, tau, and vascular pathologies can also be factors in the pathogenesis of the condition. Although the data from the current study do not present the underlying mechanisms of more severe thinning of the mGCIPL in cases of cognitive impairment in patients with PD, the occurrence might be the result of transsynaptic degeneration from cortical and subcortical neuronal loss or a common pathogenesis that underlies the association between retinal thinning and dementia^39^. It is well known that retinal thinning in dementia is caused by the intracellular deposition of amyloid-β in the inner retinal layers. Retinal thinning and decrease in the dopamine levels in PD are associated with the deposition of pathologic α‐synuclein in the retina, which is interpreted as mirroring the burden of cerebral α‐synuclein and the resultant cognitive changes of PD^40^.

Another important observation of the current study was the significant correlation between the parameters of thickness of the mGCIPL and the volumetric parameters of several brain structures (cortical GM, cortical WM, thalamus, putamen, pallidum, and hippocampus). Duncan *et al*.^41^ previously demonstrated that altered MRI measures of the integrity of cortical WM and volume of GM correlated with a cognitive decline. In addition, one study reported that patients with PD who had cognitive impairment showed reduced volumes of the thalamus and putamen^38^. Recently, an association was documented between decreased volume of the thalamus, putamen, hippocampus, and cortical GM and worse cognitive performance in patients with PD^42^. Hence, the findings of the current study provide evidence supporting the association between the parameters of the mGCIPL and cognitive impairment in patients with PD.

This study had several limitations. First, the PD group mainly consisted of patients at relatively early stages of the condition, since the quality of OCT images is often impaired in patients at an advanced stage. Thus, there was a possibility of exclusion of potential study subjects. Second, only half of the patients with PD were included in the further neuroimaging analyses, which could potentially impose a selection bias. However, the demographic characteristics and the disease severity were not significantly different between the patients who were included and those who were not included in the MRI analysis (Supplementary Table S1). Hence, we do not believe that it has influenced our study results. Third, the control subjects did not undergo neurologic examinations and brain MRI evaluation, so we cannot determine whether the relationship between the OCT parameters and neurologic examinations and volumetric data of brain MRI can be generalized to subjects other than patients with PD. We believe that it is one of the limitations that requires more investigation. We believe that in order to determine the roles of SD-OCT in PD patients, there is a need for further comprehensive study encompassing comparison of control data. Fourth, only one eye was randomly selected for the analysis in this study. Some recent studies suggest asymmetrical involvement of the retina in PD patients^43^. Therefore, incorporation of only one randomly selected eye could have influenced our study results. However, the association between the two eyes of each subjects in mGCIPL thickness was highly correlated, hence, we think that this effect might be negligible (Supplementary Table S2). Finally, the cause-and-effect relationship cannot be ascertained owing to the cross-sectional nature of the study. Longitudinal OCT data might potentiate the findings of the current study.

In conclusion, reduced thicknesses of the inferior and temporal pRNFL and the overall mGCIPL was observed in patients with PD compared to controls. The thinning was more prominent in the measurements of the mGCIPL. Moreover, the thinning of the mGCIPL was significantly associated with the cognitive function and volumetric parameters of associated brain structures. The findings of the current study add new perspectives to our understanding of the role of OCT in patients with PD, and highlight the clinical implications as a candidate biomarker for early detection of cognitive impairment in patients with PD. Future longitudinal follow-up studies are needed to validate the usefulness of OCT parameters for monitoring of patients with PD in daily clinical practice.

## Methods

### Subjects

The Institutional Review Board of Chonnam National University Hospital approved this prospective, cross-sectional study. The study followed the tenets of the Declaration of Helsinki. All patients provided written informed consent prior to enrollment in the study.

Patients were recruited from the Movement Disorders Clinic of Chonnam National University Hospital from April 2016 to December 2017. Parkinsonism was defined as the presence of bradykinesia, plus one additional clinical sign: rigidity, 4–6-Hz resting tremor, or postural instability not caused by primary visual, vestibular, cerebellar, or proprioceptive dysfunction. The patients were diagnosed with PD according to the clinical diagnostic criteria of the United Kingdom Parkinson’s Disease Society Brain Bank^44^. Diagnosis of PD was made by a movement disorder specialist (S.M.C). Only drug-naïve patients with *de novo* PD were enrolled. None of the patients had any present or past history of therapy with antiparkinsonian agents and none were taking medications with known anti-dopaminergic effects. Patients with an unclear diagnosis, atypical or secondary (such as vascular and drug-induced) parkinsonism, dementia, any severe comorbidity that may have interfered with the daily functioning (except parkinsonism), any clinically significant lesions visible on brain MRI, and those who were unable to complete the clinical evaluations were excluded.

Patients subsequently underwent a complete ophthalmic examination, including measurement of IOP using Goldmann applanation tonometry, BCVA, manifest refraction, slit-lamp examination, ONH, and RNFL examination using color stereoscopic disc photography and red-free RNFL fundus photography. Axial length and central corneal thickness were measured using optical low-coherence reflectometry (Lenstar; Haag-Streit AG, Koeniz, Switzerland). A detailed medical history was also recorded for each subject. Eyes with glaucoma, retinal pathology, such as age-related macular degeneration, diabetic retinopathy, epiretinal membrane, and retinal vein or artery occlusion, presence of media opacity, high myopia (SE refractive error greater than −6.0 D), history of prior intraocular surgery or laser treatment except simple cataract extraction, history of ocular trauma, and family history of glaucoma were excluded. Fifty-three age and sex matched healthy controls without PD were enrolled from the general eye clinic of Chonnam National University Hospital. None of the control subjects had a history or evidence of a neurologic disease of any nature. For all subjects in the control group, history was carefully obtained and subjects were strictly excluded if any suspicious past history or neurologic symptoms were observed. Each eye was considered separately, and in cases where both eyes of a subject met the inclusion criteria, only one eye was randomly selected for the study.

### Neurologic evaluation

Detailed clinical history and neurological examination was performed in all patients. Disease severity was evaluated according to the mHY scale, which is widely used to quantify the progression of symptoms of PD and categorize the patients^13^. Higher rates signify an increased severity of the disease. Parkinsonism was assessed by the UPDRS parts I (mentation, behavior and mood; score range 0–16; higher = worse), II (activities of daily living; score range 0–52; higher = worse), and III (motor function; score range 0–108; higher = worse)^14^.

The total burden of non-motor symptoms was assessed with the NMSS, which is a 30-item scale developed for the assessment of non-motor symptoms in patients with PD and consists of nine domains (cardiovascular, sleep/fatigue, mood/cognition, perceptual problems, attention/memory, gastrointestinal tract, urinary, sexual function, and miscellaneous). Each item is scored based on both severity (0–3, higher = worse) and frequency (1–4, higher = worse) of the symptoms over the past four weeks, which are then multiplied, resulting in scores for each item/domain^45^. In addition, the severity of depression in the included patients was evaluated using the Beck Depression Inventory (BDI). General cognition was assessed using the K-MMSE and MoCA tests. The MoCA is a brief, 10-minute assessment that includes questions to evaluate the cognitive domains most affected in PD, including executive function, attention, working memory, delayed recall memory, visuospatial, and language. MoCA is used as a screening tool for testing cognitive impairment in patients with PD^45,46^. The Movement Disorder Society task force on PD-MCI has recommended the MoCA as an abbreviated Level 1 diagnostic tool for possible MCI in patients with PD^47,48^. In the current study, the patients were further categorized as those with a normal cognition (PD-NC), if their MoCA scores were 26–30, or cognitive impairment (PD-CI), if their scores were ≤25, because the MoCA is generally believed to be more sensitive in diagnosing cognitive impairment than the MMSE^18,19^. The quality of life in patients with PD was evaluated using the Parkinson’s Disease Questionnaire-8 (PDQ-8).

### Spectral-domain optical coherence tomography imaging

SD-OCT images were obtained using Cirrus SD-OCT (Carl Zeiss Meditec Inc., Dublin, CA, USA) software (v.6.0). All OCT examinations were performed by the same operator who was experienced in taking OCT images. The Optic Disc Cube 200 × 200 protocol provides the results obtained from 200 horizontal B-scans (200 A-scans per B-scan) and measures pRNFL thickness in a cube of 6 × 6 × 2 mm. The average pRNFL thickness was measured within a 3.46-mm-diameter circle, the center of which was manually positioned at the optic disc center. Quadrant (superior, inferior, nasal, and temporal) pRNFL thickness values were used in our analysis.

The Macular Cube 512 × 128 scan which covers an area of 6 × 6 mm of the retina provides the results for the whole retinal and mGCIPL thickness. The center of the macula was set as the center, and the retinal map analysis system was used to divide the macula into a central circle, inner ring, and outer ring with diameters of 1, 3, and 6 mm, respectively. The OCT software determined the retinal thickness as the distance between the vitreoretinal interface and the hyperreflective band corresponding to the retinal pigment epithelium. The central foveal subfield was bounded by the innermost 1-mm diameter circle. The CFT, overall average MT, and overall MV over the entire grid area were obtained from the computational software output. The ganglion cell analysis algorithm in the 6.0 software version of Cirrus OCT reports the combined thickness of the retinal ganglion cell and inner plexiform layers by identifying the outer boundaries of the RNFL and the inner plexiform layer. The average, minimum (lowest mGCIPL thickness over a single meridian crossing the annulus), and six sectoral (superotemporal, superior, superonasal, inferonasal, inferior, and inferotemporal) mGCIPL thicknesses were measured within a 14.13 mm^2^ elliptical annulus area (vertical inner and outer radii of 0.5 mm and 2.0 mm, respectively, horizontal inner and outer radii of 0.6 and 2.4 mm, respectively) centered on the fovea within the cube. All OCT scans were reviewed for image quality and excluded from the analysis in cases of signal strength less than 7, decenteration of the pRNFL sampling circle, segmentation errors, or motion artifacts defined as a discontinuity of the blood vessels.

### MR imaging scanning and image processing

Some of the patients with PD previously underwent brain MRI at various outpatient or hospital-based institutions and additional brain MRI were not indicated in such patients. MRI scans, including 3D volumetric scans, were newly acquired from 37 patients at the Chonnam National University Hospital. To limit the variations caused by instrument-related factors, only those MRIs taken at the Chonnam National University Hospital were selected for the analysis.

MRI examinations were conducted using a 1.5-Tesla (T) GE Signa HDxt scanner (General Electric, Milwaukee, Wisc., USA). The participants underwent MRI with a 3D sagittal T1-weighted SPGR (spoiled gradient recalled acquisition: TR = 9.9 ms, TE = 4.0 ms; flip angle = 13°; FOV = 240 mm; slice thickness = 1.2 mm with no gap; NEX = 1; acquisition matrix = 288 × 192), axial T2-weighted, and FLAIR images to obtain 130 images covering the entire brain. FLAIR and T2-weighted images were used to detect abnormal ventricular dilatation and white matter lesions.

MRI data were processed using FreeSurfer v6.0.0 (http://surfer.nmr.mgh.harvard.edu), which is a set of automated tools for segmentation and surface reconstruction of the brain from structural MRI data. All image-processing steps were performed by one imaging expert (B.C.K) who was blinded to all the clinical information. The 3D image files were transferred in the DICOM format to a macOS (High Sierra v10.13.3) workstation for morphometric analysis. The subcortical volume was measured automatically using FreeSurfer. The automated procedures for volumetric measurements of the different brain structures have been described previously^16,17^. This procedure automatically provided segments and labels for ≤40 unique structures and assigned a neuroanatomic label to each voxel in an MR imaging volume on the basis of probabilistic information estimated automatically from a manually labeled training set. The segmentation was visually inspected for accuracy and correct segmentation was ascertained. Any geometric inaccuracies or topological defects were corrected using a combination of automatic and manual methods. Confirmation of the segmentation and manual editing was performed by an imaging expert (B.C.K). Calculation of total intracranial volume was done using FreeSurfer, as described previously^49^. The volumes of all cortical and subcortical structures were adjusted for the total intracranial volume in each patient. Example of FreeSurfer segmentation is provided in Supplementary Figure S1.

### Statistical analyses

SPSS version 19.0 (SPSS Inc., Chicago, IL) was used for the statistical analyses. The normality of distribution was verified using the Shapiro-Wilk normality test, and parametric or nonparametric tests were subsequently used. The baseline characteristics were reported as counts and proportions or mean ± SD values, as appropriate. The differences in demographic features and OCT-determined parameters between the patients and healthy controls were compared using the chi-square or independent *t* test. Comparisons between the PD-NC and PD-CI groups were performed using the independent *t* or Mann-Whitney *U* tests, as appropriate. Pearson’s correlation analysis was used to determine the correlation coefficients of the various clinical grading scales and OCT-determined parameters in patients with PD. ROC curves were plotted and the AUC was obtained to determine the accuracy of the OCT parameters to detect possible cognitive impairment in patients with PD. Statistical significance was set at *P* < 0.05.

### Data Availability

The datasets generated during the current study are available from the corresponding author on reasonable request.

## Supplementary information

Supplementary Information
