# Supplementary material for: Inner retinal thinning as a biomarker for cognitive impairment in de novo Parkinson’s disease
Source: Sci Rep. 2019 Aug 14;9:11832. doi: 10.1038/s41598-019-48388-7 (PMC6694167; doi:10.1038/s41598-019-48388-7)

**Inner retinal thinning as a biomarker for cognitive impairment in *de novo* Parkinson’s disease**

Mi Sun Sung,^1†^ Seong-Min Choi,^2†^ Jonghwa Kim,^1^ Jun Young Ha,^1^Byeong-Chae Kim,^2^ Hwan Heo,^1^

and Sang Woo Park^1*^

^1^Department of Ophthalmology, Chonnam National University Medical School and Hospital, Gwangju, South Korea

^2^ Department of Neurology, Chonnam National University Medical School and Hospital, Gwangju, South Korea

^†^These authors equally contributed to this work as first authors.

**Supplementary Table S1. Comparison of demographics between the PD patients who were included and those who were not included in the MRI analysis.**

| **Characteristics** | **PD patients included in the**  **MRI analysis**  **(n = 37)** | **PD patients not included in the**  **MRI analysis**  **(n = 37)** | ***P*-value^*^** |
| --- | --- | --- | --- |
| Age (years) | 66.05 ± 9.09 | 64.54 ± 7.67 | 0.441 |
| Sex (male : female) | 14 : 23 | 11 : 26 | 0.312 |
| BCVA (logMAR) | 0.06 ± 0.09 | 0.04 ± 0.06 | 0.436 |
| SE refractive error (D) | 0.41 ± 1.65 | 0.60 ± 1.07 | 0.560 |
| IOP (mmHg) | 14.84 ± 2.62 | 14.76 ± 3.50 | 0.910 |
| Axial Length (mm) | 23.89 ± 1.42 | 23.46 ± 0.73 | 0.129 |
| Central corneal thickness (μm) | 522.03 ± 47.45 | 529.82 ± 36.79 | 0.467 |
| Disc area (mm^2^) | 2.10 ± 0.46 | 2.12 ± 0.38 | 0.761 |
| Vertical CDR | 0.56 ± 0.14 | 0.52 ± 0.14 | 0.280 |
| Neurologic examinations |  |  |  |
| mHY scale | 1.81 ± 0.79 | 1.72 ± 0.74 | 0.598 |
| UPDRS part I | 1.56 ± 1.79 | 1.91 ± 2.35 | 0.624 |
| UPDRS part II | 6.77 ± 6.07 | 5.94 ± 4.24 | 0.521 |
| UPDRS part III | 20.00 ± 8.72 | 17.57 ± 8.70 | 0.234 |
| NMSS | 44.88 ± 38.31 | 41.11 ± 31.43 | 0.656 |
| BDI | 35.71 ± 11.76 | 32.40 ± 14.62 | 0.305 |
| PDQ-8 | 6.60 ± 6.37 | 3.73 ± 3.37 | 0.126 |
| K-MMSE | 25.38 ± 3.02 | 26.00 ± 3.94 | 0.449 |
| MoCA | 23.34 ± 4.63 | 24.40 ± 5.12 | 0.381 |

PD = Parkinson’s disease; MRI = magnetic resonance imaging; BCVA = best-corrected visual acuity; SE = spherical equivalent; D = diopters; IOP = intraocular pressure; CDR = cup-to-disc ratio; mHY = modified Hoehn & Yahr scale; UPDRS = United Parkinson’s Disease Rating Scale; NMSS = Non-Motor Symptom Scale; BDI = Beck Depression Inventory; PDQ-8 = Parkinson’s disease quality of life 8 questions; K-MMSE = Korea version of the Mini-Mental State Examination; MoCA = Montreal Cognitive Assessment.

Data are mean ± standard deviation unless otherwise indicated.

**^*^** *P*-values derived from Student-*t* test or Chi-square test as appropriate.

**Supplementary Table S2. Correlation between the two eyes of each PD patients in mGCIPL thickness.**

|  | **r** | ***P*-value** |
| --- | --- | --- |
| OD Minimum vs. OS Minimum | 0.450 | 0.001 |
| OD Average vs. OS Average | 0.637 | < 0.001 |
| OD Superonasal vs. OS Superonasal | 0.585 | < 0.001 |
| OD Superior vs. OS Superior | 0.533 | < 0.001 |
| OD Superotemporal vs. OS Superotemporal | 0.554 | < 0.001 |
| OD Inferotemporal vs. OS Inferotemporal | 0.510 | < 0.001 |
| OD Inferior vs. OS Inferior | 0.591 | < 0.001 |
| OD Inferonasal vs. OS Inferonasal | 0.554 | < 0.001 |

**Supplementary Figure S1.** Example of FreeSurfer segmentation. 3D volumes were segmented using FreeSurfer v6.0.0.


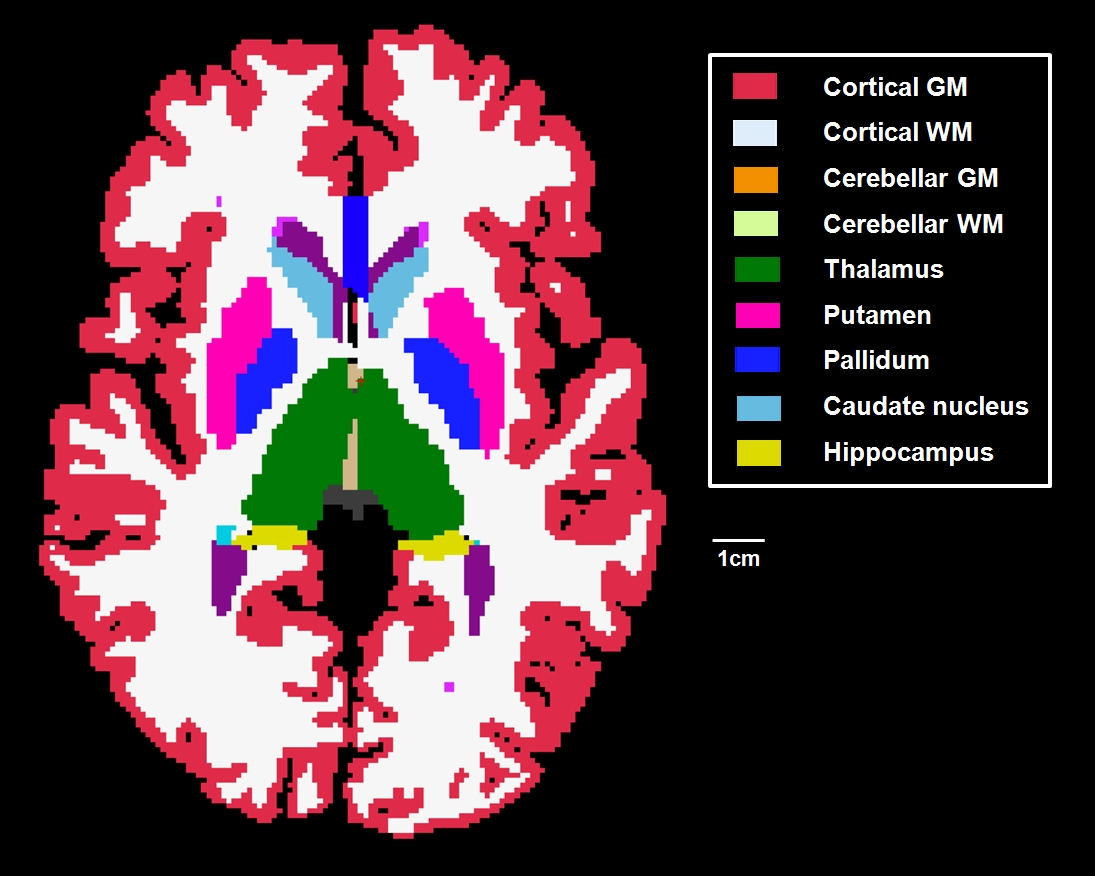

Supplement: Supplementary file 1 — Supplementary Information [file 41598_2019_48388_MOESM1_ESM.docx]
